# Real-Time Multi-Person Video Synthesis with Controllable Prior-Guided Matting

**DOI:** 10.3390/s24092795

**Published:** 2024-04-27

**Authors:** Aoran Chen, Hai Huang, Yueyan Zhu, Junsheng Xue

**Affiliations:** School of Information and Communication Engineering, Beijing University of Posts and Telecommunications, Beijing 100876, China; car13021950113@bupt.edu.cn (A.C.); bupt_xjs@bupt.edu.cn (J.X.)

**Keywords:** deep learning, video matting, controllable information, deep guided filter

## Abstract

In order to enhance the matting performance in multi-person dynamic scenarios, we introduce a robust, real-time, high-resolution, and controllable human video matting method that achieves state of the art on all metrics. Unlike most existing methods that perform video matting frame by frame as independent images, we design a unified architecture using a controllable generation model to solve the problem of the lack of overall semantic information in multi-person video. Our method, called ControlMatting, uses an independent recurrent architecture to exploit temporal information in videos and achieves significant improvements in temporal coherence and detailed matting quality. ControlMatting adopts a mixed training strategy comprised of matting and a semantic segmentation dataset, which effectively improves the semantic understanding ability of the model. Furthermore, we propose a novel deep learning-based image filter algorithm that enforces our detailed augmentation ability on both matting and segmentation objectives. Our experiments have proved that prior information about the human body from the image itself can effectively combat the defect masking problem caused by complex dynamic scenarios with multiple people.

## 1. Introduction

**Figure 1:**
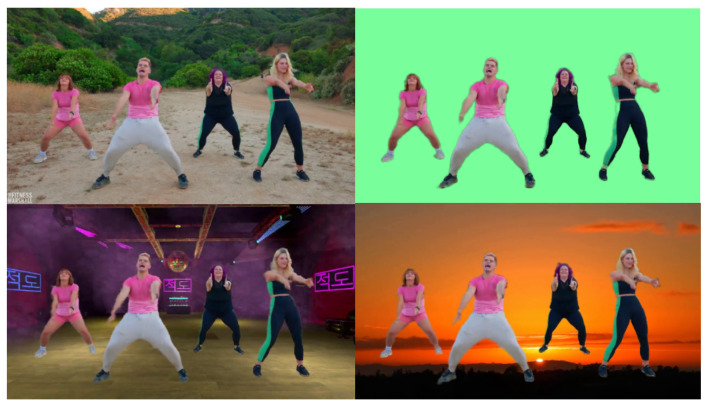
An application demonstration of video synthesis using a multi-person matting algorithm.

As a classical image processing task, the matting algorithm for image and video has been widely applied in image editing software, entertainment video creation, and web teleconferencing. Real-time image matting [1,2] and video matting algorithms [3] based on deep learning have been extensively studied. Figure 1 shows a typical video synthesis application. Similar to the semantic segmentation task, the matting algorithm needs to extract the edge probability priors and feature patterns contained in specific semantic categories from the global frames. We can define the matting equation for single frame *I* as the linear combination of foreground *F* and background *B* through mask α as follows:

(1) I=α×F+(1−α)×B,α∈[0,1].

The direct search in the solution space of the indeterminate equation is quite difficult because the high-resolution frame input corresponds to a very high-dimensional feature space. The solution of foreground with green-screen props can also be seen as a simple case where a strong prior distribution has been known [4]. If we regard the matting task as a downstream task of the generative model, using a generative adversarial network or a large-parameter generative model such as a diffusion model [5,6] can directly obtain a sufficiently realistic mask. However, the controllability and real-time performance of the generative model are difficult to guarantee, and it cannot replace the existing fast and robust matting model in terms of real-time and practicality.

Some matting models processed frame by frame are not always satisfactory and generate artifacts in complex multi-person videos. Our research focuses on combining the advantages of large generative models [7] and lightweight video salient object detection models [8,9,10] to improve the matting quality. Most matting approaches neglect the most available feature in videos: human body semantics. For instance, in segmentation tasks, any other auxiliary information is not prerequisite when images are taken naturally and contain clear object relationships.

Human body semantics information can also be easily extracted via these clear object relationships and improve the matting performance in many ways [11,12,13]. First, human body information can assist the model in locating key positions such as the head and limbs of the human body. The performance of matting mainly depends on whether the mask of the human body is complete. The model should optimize the details such as edge hair after the human body is correctly segmented. Second, the introduction of human motion information helps to enhance the inter-frame stability of the matting results. As temporal information can improve matting robustness, it can also help the model understand the occluded and revealed background. In practical applications, video frames are not typically transmitted individually or as a complete video stream. Instead, they are transmitted in packets, necessitating the model’s ability to extract useful dynamic information from a limited number of frames. In this case, the estimation of human motion posture will be more effective and accurate than the motion prediction of the entire image. Third, human body information can help the model better distinguish the dynamic background. Although background priors in videos are often unknown and difficult to obtain, there have been many successful works on the understanding of semantic priors on key points of human poses [11,14]. When the position of the human body in the area has been roughly positioned, the interference to the model caused by the complex video background will be greatly reduced. Therefore, we propose a controllable matting architecture to exploit the controllable prior information of human bodies. Our method significantly improves the foreground alpha quality and has better interpretability.

The matting performance of high-resolution video frames is closely related to the degree of restoration of edge details. Similar to super-resolution algorithms [15,16,17], existing deep learning models with an encoder–decoder structure need to learn stronger pixel-level semantic restoration abilities, from low-resolution features to high-resolution features. For tasks related to the human body, the main difficulty is in capturing the semantics of scattered hair edges and clothes corners far away from the human body. Coarse-to-fine or refinement structure [18] are methods commonly used to obtain a lower upsampling error. Therefore, we propose a detailed refiner module in the decoder based on a learnable multi-level image filter, which decouples decoder features, filter features, and fine-tuning masks to achieve separation of the overall segmentation task and the edge detail enhancement task.

A scenario that will cause most matting models to fail is matting on multi-person dynamic videos, such as some augmented reality applications, where the model often outputs defective masks due to an insufficient ability to capture semantic information. Some methods use semantic segmentation models to preserve the integrity of masks but introduce an additional training burden and slow down the inference. A feasible solution is to introduce controllable conditional priors commonly used in generative models and introduce the obvious human visual information in the image to prompt semantic information instead of mandatory semantic segmentation. Furthermore, we propose a new deep learning-based image filter algorithm to enforce our detail augmentation ability on both matting and semantic segmentation objectives simultaneously. Training with a segmentation objective can also effectively regulate our model without additional adaptation steps. Benefiting from the sharpening of semantic information and the filtering of detailed information, our method outperforms the previous state-of-the-art method while being very light and fast. We summarize our contributions as follows:

- We design a controllable encoder to build the recurrent matting model with generative architecture. Controllable human body prior information can obviously preserve the quality of alphas in complex, dynamic, multi-person videos.
- We propose a learnable and mask-guided image filter algorithm to augment the edge detail of each human alpha, which can effectively improve the video matting quality.
- We evaluate our ControlMatting model on the VM and AIM datasets, and experimental results prove that ControlMatting can achieve the state of the art on all metrics. Compared with the frame-by-frame-based matting model, we consistently obtain significant improvements.

## 2. Related Work

### 2.1. Classical and Trimap-Based Matting

Green screen matting is a classical matting method when the background is close to a solid color, but the green edge needs image blending in post-processing [4,19]. Trimap labels are used to solve the unknown regions in the non-solid color background in non-learning and deep network methods [20,21]. Trimap is easier to obtain manually, and separating the foreground, background, and unknown regions helps the model focus on edge information extraction in unknown regions for an anonymous object. While feature maps extracted from trimap labels are not sufficient, InteractiveMatting [22] and UnsupervisedMatting [23] choose to utilize additional information, such as manual interaction information or pixel-level clustering patterns. Auxiliary modules based on the attention mechanism [24] and independent mask processing modules [25] are also widely used in image matting models to improve the ability of global feature capturing. To extend target guidance to video, Sun et al. proposed DVM [26], which uses simple convolutional layers to align local spatial information. GFM [27] decomposes the task into two parallel sub-tasks—high-level semantic segmentation and low-level detail matting—and employs a shared encoder and two separate decoders to learn both tasks in a collaborative manner for end-to-end natural image matting. BGM [4] proposes background matting, which includes a switching prior encoder to learn background context. BGMv2 [1] has been proposed for the requirement of real-time and high-resolution matting. TIMI-Net [28] independently designed the RGB-unit and the Trimap-unit to realize tripartite information mining and integration. TransMatte [29] redesigned the trimap as three learnable tri-tokens for introducing advanced semantic features into the self-attention mechanism. MatteFormer [30] computes prior tokens of the transformer backbone, which outperformed convolutional models on low-resolution images. However, trimap-based matting cannot handle complex dynamic backgrounds and moving shots.

### 2.2. Auxiliary-Free Video Matting

Portrait matting has higher fineness of edge details, and automatic matting without auxiliary information has been researched [31,32]. Obtaining a dataset with the overall trimap labels of the video is always difficult, as only a few frames of trimap are often marked for auxiliary video matting [33]. P3M-Net [27] designs a unified framework that utilizes semantic awareness and detail matting, with special emphasis on their interaction with the encoder to facilitate the matting process. MODNet [2] proposes a postprocessing trick that compares the prediction of neighboring frames to suppress flicker, but it cannot handle fast-moving body parts, and the model itself still operates on frames as independent images. ModNet separates overall semantic positioning and detail information restoration into two tasks, and the learning of detailed information [34] has always been emphasized in auxiliary-free matting tasks. PP-Matting [35] learns the semantic map and the detail map by guidance flow to fuse the refined matte alpha. VideoMatt [36] proposes a new benchmark for video matting algorithms to better weigh matting speed and quality, but the model structure evaluated is too simple. AdapM [37] designs an adaptive interconnected framework for simultaneously differentiating foregrounds from backgrounds and capturing alpha matte details of human subjects in the foreground to eliminate trimap dependency. The latest real-time high-quality model is RVM [3], which adopts recurrent architecture as the strategy for joint training on semantic segmentation datasets. An edge detail augmentation approach uses Deep Guided Filter (DGF) [38], a post-processing filter that introduces high-resolution frames as guidance and directly uses a learnable convolutional layer instead of solving the window matrix. On the contrary, our method focuses on using controllable prior information from human body features to improve the matting quality in multi-person dynamic video.

### 2.3. Instance Segmentation and VSOD

Previous works have explored using recurrent architectures for instance segmentation tasks [39] and video salient object detection (VSOD) tasks [8,40,41]. Instance segmentation tasks and VSOD tasks can also be completed simultaneously through cross-referencing by a unified segmentation framework [42]. The matting task is more about how to obtain more accurate edge details, but some methods directly superimpose the detailed post-processing module on the instance segmentation or VSOD model for better temporal robustness. Patch-based refinement has been explored by PointRend [43] for segmentation and BGMv2 [1] for matting, but the quality of motion cannot be guaranteed [44,45]. TCR-Net [46] achieves deep feature extraction by a pure vision transformer with multi-resolution token representations to integrate appearance and motion. DynamicVSOD [6] adopts a multi-path fusion branch structure to improve the temporal robustness of each prediction salient mask. However, these models only perform convolutional operations on selective patches to reduce the computational burden brought by the temporal module. Recurrent matting models have shown improved performance compared to their image-based counterparts.

### 2.4. Generative Model

Previous works have explored using GAN for image matting and benchmark discrimination [47,48]. SM-GAN [49] proposes a dual hierarchical aggregation network to simultaneously complete the matting task and the background restoration generation task. OmniMatte [50] estimates alpha mattes and color images of objects including shadows, reflections, and generated smoke, as well as all their associated time-varying scene elements. FactorMatte [51] maps objects at different levels in the video into independent factors and uses a Bayesian framework to decompose complex conditional interactions belonging to objects between different layers. OmniMatte and FactorMatte employ multimodal learning to train matting models, utilizing modal information other than the original video to assist in multifunctional matting. Auxiliary modalities include shadows, optical flow, explicit smoke, and video depth information, and modalities impose higher demands on model capacity, limiting the effectiveness on lightweight models with small parameter counts. Additionally, when the model only has access to the original data of the image or video, it cannot immediately obtain other auxiliary information. This explicit multimodal information is not sufficiently controllable. Therefore, in designing the controllable encoder for extracting conditional prior information, our model integrates additional modal information as reference features rather than using them as mandatory constraint types. Diffusion models have achieved dominance [7,52] in generative domains and can be used to tackle other vision domain tasks as well [5,53]. ControlNet [54] firstly proposes that controllable prior information can be added into a generative model to constrain the generation paradigm. However, a computationally intensive model is not suitable for the mobile matting task, so our model introduces guiding auxiliary prior information but does not need to solve the diffusion equation.

## 3. Methods

### 3.1. Network Architecture

**Figure 2:**
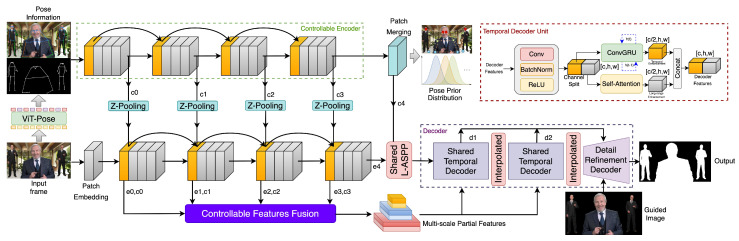
Overall architecture of our ControlMatting model.

Our architecture consists of a four-stage encoder that extracts fundamental features in each frame, a controllable encoder that incrementally learns specific information, and a shared decoder with detailed filter module for recurrent memory and detail enhancement. Figure 2 shows our network architecture. In the matting task, the encoder needs the ability to locate objects accurately, and our encoder follows the design in FasterNet [55], which has lower FLOPS and better classification performance. Although the encoder with downsampling convolution has been widely applied in salient object detection and segment tasks, we change the 1/4 downsampling layer in FasterNet to 1/2 downsampling and call this FasterNet* because 1/4 downsampling features may lose low-level information and pose a challenge for dilated layers in the decoder to recover edge details.

The controllable encoder is connected to the fundamental encoder by selector modules to produce strong auxiliary prior information, and it also adopts FasterNet* as the backbone. The features of the controllable encoder are weighted and fused with the feature maps of the base encoder after passing through vertical Z-Pooling modules. These Z-Pooling layers consist of 1 × 1 convolutional layers, all initialized to 0, serving as feature channel selectors. Before entering the shared temporal decoder unit, the final output of the encoder undergoes multi-scale feature integration, which is achieved by the L-ASPP layer. The L-ASPP module is proposed in RVM [3] to save inference time and is adopted to extract multi-scale feature maps of the final output in encoder Stage 4.

In the shared decoder, we introduce ConvGRU behind the upsampling layer to keep it temporally robust, and we propose a progressive refined filter with pixel-level mask-guided refiner module to further recover the edge details for high-resolution frames. The temporal decoder unit in the network comprises convolutional modules and a parallel ConvGRU unit along with a self-attention module, which achieves temporal feature extraction by splitting feature channels and learns global long-range attention dependencies. From these two aspects, the temporal decoder unit completes the semantic decoding of temporal feature information, providing semantically clear low-resolution coarse mask results for subsequent progressively refined filters to obtain the final matting results.

### 3.2. Controllable Encoder

The purpose of the controllable encoder is to provide the decoder with useful conditional prior information and implicit human body area positioning guidance so that the model can more accurately divide the foreground and background areas when the difference in color distribution between the foreground and the background is small, and to separate the semantic information belonging to different human bodies so that, when the model handles complex multi-person occlusion scenes, it will not be misled by the occluded limbs, leading to erroneous semantic understanding. The controllable encoder adopts the same architecture of the fundamental encoder and learns only conditionals prior to replenishing the semantic structure. Conditional prior information can manipulate the input priors of the decoder blocks so as to further control the overall behavior of the entire network because of the backpropagation. Specifically, given an input tensor $x\in R^{h\times w\times c}$, a convolutional block F(·,·) in an encoder with a set of parameters $\left\{\omega_{i},b_{i}|\Theta_{i}\right\}$ can transform *x* into the extracted feature map *y* by

(2) $$y_{i}=F_{i}\left(x,\left\{\omega_{i},b_{i}|\Theta_{i}\right\}\right),$$

where *i* denotes the *i*th stage in the encoder. In order to aggregate the conditional prior vector $C\in R^{h_{c}\times w_{c}\times c_{c}}$, we introduce selector modules to learn the salient distribution of conditional priors, called the Z-Pooling layer. Selector modules consist of several 1 × 1 convolution layers, and both weight and bias are initialized with zeros to avoid the negative effects of random initialization. We denote the selector module as S(·,·) and use $\left\{\omega_{ic},b_{ic}|\Theta_{ic}\right\}$ to describe the calculation process as follows:

(3) $$\begin{array}{cc} y_{0} & =F(x_{0}+S\left(C,\left\{\omega_{0c},b_{0c}|\Theta_{0c}\right\}\right))\\ y_{ic} & =y_{i}+S\left(y_{0},\left\{\omega_{ic},b_{ic}|\Theta_{ic}\right\}\right), \end{array}$$

where $y_{ic}$ works as the conditional prior feature of each stage in the whole encoder. In the training step, we get

(4) $$\begin{array}{cc} y_{0,step=1} & =F\left(x_{0}+S\left(C,\left\{\omega_{0c},b_{0c}|\Theta_{0c}\right\}\right)\right)=F{\left(x_{0}\right)}_{step=1}\\ y_{ic,step=1} & =y_{i}+S\left(F\left(x_{0}\right),\left\{\omega_{ic},b_{ic}|\Theta_{ic}\right\}\right)=y_{i,step=1} \end{array},$$

which means the controllable encoder works as auxiliary fine tuning when training epochs are not large and can preserve the capability and result quality of the fundamental matting model. For a single 1 × 1 convolution layer, the forward pass can be written as follows:

(5) $$\begin{array}{cc} S\left(x,\left\{\omega_{c},b_{c}|\Theta_{c}\right\}\right){|}_{\Theta_{c}=0} & =b_{c}+\sum_{p}^{c}\omega_{c}\left(p\right)x\left(p^{*}\right), \end{array}$$

where *p* denotes the iterator spatial position, and $p^{*}$ denotes the selected channel index. In the first gradient descent iteration, we have

(6) $$\begin{array}{cc} \partial L_{c} & =\frac{\partial L}{\partial S_{i}}\times \frac{\partial S_{i}}{\partial \omega_{ci}}+\frac{\partial L}{\partial S_{i}}\times \frac{\partial S_{i}}{\partial b_{ci}}+\frac{\partial L}{\partial S_{i}}\times \frac{\partial S_{i}}{\partial x_{i}}\\ \; & =\frac{\partial L}{\partial S_{i}}\times \frac{\partial S_{i}\left(x_{i},\left\{\omega_{ci},b_{ci}|\Theta_{c}\right\}\right)}{\partial \omega_{ci}}+0{{|}_{w_{ci}=0}+0|}_{b_{ci}=0}\\ \; & =\frac{\partial L}{\partial S_{i}}\times \frac{\partial \sum_{p}^{c}\omega_{ci}\left(p\right)x_{i}\left(p^{*}\right)}{\partial \omega_{ci}}\\ \; & =\frac{\partial L}{\partial S_{i}}\times x_{i}\left(p^{*}\right)\neq 0 \end{array}$$

in the first step, where $L_{c}$ denotes the loss function when the model trains the controllable encoder. In the steps after that, $\omega_{ci}$ will be learned and obtain non-zero gradients, such that the second term of the loss function is also non-zero. In this way, the controllable encoder optimizes its parameters step by step and can be fine-tuned as an auxiliary subnetwork.

### 3.3. Progressive Refined Filter

**Figure 3:**
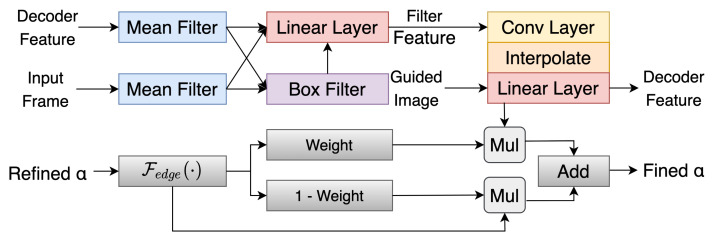
The structure of our detailed refiner decoder with progressively refined filter.

The biggest difference between the matting task and the segmentation task lies in the different granularity requirements for the edge details. Compared to the instance segmentation model, the matting model needs to transform the rough edges into finer edges. We chose the image filter to achieve this task as in the deep guided filter, and we propose a trainable filter layer with a mask-guided module, as shown in Figure 3.

Different from interpolation algorithms, traditional image filters can be viewed as calculating multiple linear transformation windows by setting a predetermined distribution form. Obtaining adaptive pre-distribution estimates of edges in an image is often difficult, but these parameters can be obtained from other places, such as high-resolution guide maps and their edge features or learnable decoder features. For a normal filter window $W_{k}$ and an input image $I_{i}$, we have the output of the filter as follows:

(7) $$O_{i}={\left|W\right|}^{-1}\sum_{i\in W_{k}}\left(a_{k}*I_{i}+b_{k}\right),$$

where *W* denotes the learnable parameters of the filter window, and $\left\{a_{k},b_{k}\right\}$ denote the linear transformation parameters. When given guided image $G_{i}$, we build the loss function as follows:

(8) $$L\left(a_{k},b_{k}\right)=\sum_{i\in W_{k}}{\left(a_{k}I_{i}+b_{k}-G_{i}\right)}^{2}+\epsilon a_{k}^{2},$$

which can be solved as:

(9) $$\begin{array}{cc} a_{k} & ={\left(\Sigma_{k}+\epsilon \right)}^{-1}{\left|W\right|}^{-1}\sum \left(I_{i}G_{i}-\mu \left(I_{i}\right)\mu \left(G_{i}\right)\right),\\ b_{k} & =G_{i}-a_{k}I_{i}, \end{array}$$

where μ(·) denotes the mean filtering layer. In Figure 3, the filter layer works when upsampling and learning edge-preserving transformation, and both the guided image $G_{i}$ and the guided mask are introduced to build a progressive refined filter. The predicted result of each layer in the decoder can be written as follows:

(10) $$\left(y_{i},\alpha_{i}\right)=a_{i,k}F\left(x_{i}\right)+b_{i,k},$$

where $\alpha_{i}$ denotes the predicted alpha mask, but values of the elements in $\alpha_{i}$ are distributed between 0 and 1. Here, we chose to extract the indeterminate region by upsampling layer $S_{th}$ with threshold selecting and to introduce the guided mask map $\alpha_{i}$ to further increase the detailed information as follows:

(11) $$\alpha_{i,k}=S_{th}\left(\alpha_{i-1,k}\right)\alpha_{i}+\left(1-S_{th}\left(\alpha_{i-1,k}\right)\right)\alpha_{i-1,k},$$

which is similar to the form of the matting equation, but where the alpha mask becomes the posterior variable. The threshold selecting can preserve the confidence regions predicted from higher-level features, while the refining in uncertain regions can learn more precise details. We adopt a convolution layer to solve the $a_{k}$ instead of explicit matrix operations as follows:

(12) $$\begin{array}{cc} a_{i,k} & =S_{hid}\left(x_{i}*G_{i,l},\mu \left(x_{i}\right)*\mu \left(G_{i,l}\right),y_{i-1},\alpha_{i-1}\right), \end{array}$$

where $S_{hid}$ concatenates the input features, and the work proves that three layers of convolution optimization can fully approximate the accuracy of the image filter matrix solution. After the calculation of low-resolution filter parameter $\left\{a_{k},b_{k}\right\}$, we can obtain the high-resolution filter parameter $\left\{A_{k},B_{k}\right\}$ by

(13) $$A_{i,k}=S_{↑}\left(a_{i,k}\right),B_{k}=S_{↑}\left(b_{i,k}\right),$$

where $S_{↑}$ means the upsampling selector layer. When a high-resolution guided image is given, the output of the refined filter can be obtained by

(14) $$\left(Y_{i},A_{i}\right)=A_{i,k}*F_{h}\left(G_{i,h}\right)+B_{i,k},$$

and we define the edge region function $F_{edge}\left(\cdot \right)$ as

(15) $$F_{edge}\left(A_{i-1}^{*}\right)=\left\{\begin{array}{cc} 1 & \mathrm{if}\;0<A_{i-1}^{*}\left(\mathrm{Region}\right)<1,\\ 0 & \mathrm{otherwise}. \end{array}\right.$$

We use $F_{edge}\left(\cdot \right)$ to compute the edge-guided mask ω and $A_{i-1}^{*}$ as the previous layer, so the final output of our detailed refiner can be written as:

(16) $$A_{i}^{*}=\omega *A_{i}+\left(1-\omega \right)*A_{i-1}^{*},$$

where $A_{i}^{*}$ is the solution to the matting equation. With the mask-guided refinement, the filter can progressively learn the detailed information in different level features. The guided mask can help the trainable filter learn correct information in the initial training steps because of the edge constraints of the rough masks.

## 4. Experimental Results

### 4.1. Experimental Setting

#### 4.1.1. Dataset Information

Our model is trained on VideoMatte240K (VM) [1] and Adobe Image Matting (AIM) [56] for matting learning. VM provides 484 4K/2K video clips and corresponding high-quality alphas. AIM is an image matting dataset, and we use human images in it as the foreground composite materials. In order for the model to have better detail accuracy, we do not use the trimap-based dataset. Although the focus of the semantic segmentation task is different from that of the matting task, the rough mask in the semantic segmentation dataset is still helpful for human body positioning. As high-quality semantic segmentation datasets are readily available, the YouTubeVIS [57] and COCO [58] datasets, which are quite commonly used semantic segmentation datasets, are used for semantic information learning, and a different training strategy is adopted, as our model only learns on objects labeled “human” in the dataset.

As we introduce human pose information into our conditional encoder, the MS COCO Keypoint dataset is used for human pose estimation learning. Our model extracts human pose information by a ViTPose [11] architecture model based on the MS COCO Keypoint dataset. When we set human pose motion as controllable prior information, the key point data obtained by ViTPose are difficult to embed directly because the convolution network does not have position encoding. We connect the skeletal joint points as the binary mask for the ball-and-stick model, and the model replaces the discrete joint point coordinate data with these masks and inputs them into the conditional encoder to achieve controllable matting.

To increase the data variety and the generalization ability of the model, we apply motion and temporal augmentations, including affine translation, brightness, saturation, contrast, hue, scale, rotation, sheer, noise, and blur. In order to ensure the robustness of the model when conditional prior information is not input, we randomly delete the bounding boxes of pose estimation to simulate the failure of semantic detection of some frames in the actual application.

#### 4.1.2. Training Strategy

We propose a three-stage training strategy for the matting task to learn core semantic features and edge-refined features. We use one stage to learn sequence dependency information, one stage to learn conditional prior auxiliary information, and one stage to learn the high-resolution detail information. We use Adam as the optimizer to control the learning rate at different stages. All training is done in one NVIDIA GeForce RTX 3090 with 24 GB GPU memory.

- Stage 1: We train the base encoder, L-ASPP layer, and the shared decoder for 20 epochs. The dataset is used for matting training, and the semantic segmentation dataset is inserted intermittently in every epoch iteration. Our machine can afford 25 frames of training at the same time, so we set the sequence length as 25. The base encoder backbone is initialized by FasterNet*, and the learning rate is 1 × $10^{-4}$. Other blocks adopt 2 × $10^{-4}$ as the learning rate.
- Stage 2: Before Stage 2 training, we copy the parameters in the base encoder into a controllable encoder to save on training time. The base encoder is still trained with a small learning rate, 1 × $10^{-5}$, and the controllable encoder with a large learning rate, 1 × $10^{-4}$. Our two encoders use a unified L-ASPP middle layer and a shared decoder, with the same learning rate in Stage 1, and are trained for 5 more epochs. The semantic segmentation dataset is no longer needed.
- Stage 3: We train high-resolution videos and images here, and the VM and AIM datasets are used to create composite training frames, which need a detailed refinement decoder to recover the edge information. We train the whole model for 5 more epochs and set the base encoder learning rate as 1 × $10^{-5}$, the controllable encoder as 2 × $10^{-5}$, the detailed refiner decoder as 2 × $10^{-4}$, and the others as 1 × $10^{-5}$.

#### 4.1.3. Loss Functions

The goal of model training is to get as fine an alpha for the input frame as possible, so we adopt L1 loss to constrain the predict alpha, $\alpha^{*}$, when the ground truth, $\alpha^{t}$, is known. This one is the main loss of our model and can be written as:

(17) $$L_{1}\left(\alpha_{i}^{*},\alpha_{i}^{t}\right)=\left|\right|\alpha_{i}^{*}-\alpha_{i}^{t}{\left|\right|}_{1}.$$

A single L1 loss is quite a hard constraint in a generative procedure, so we introduce pyramid Laplacian loss, $L_{lap}\left(\cdot \right)$, to produce better optimization [59]. Lap loss can minimize the multi-scale perceptual difference by processing it through a multi-layer Gaussian kernel. This one can be written as follows:

(18) $$L_{lap}\left(\alpha_{i}^{*},\alpha_{i}^{t}\right)=\sum_{s=1}^{n}\frac{2^{s-1}}{n}\left|\right|L_{pyr}\left(\alpha_{i}^{*}\right)-L_{pyr}\left(\alpha_{i}^{t}\right){\left|\right|}_{1},$$

where *n* is the order of Laplace expansion, and in all stages we set *n* as 5. In order to ensure the inter-frame continuity of the matting performance, a timing loss constraint, $L_{T}\left(\cdot \right)$, is also necessary. We apply the temporal loss as follows:

(19) $$L_{T}\left(\alpha_{i}^{*},\alpha_{i}^{t}\right)=||\Delta \alpha_{i}^{*}-\Delta \alpha_{i}^{t}{\left|\right|}_{2}.$$

Total matting loss can be written as follows:

(20) $$\begin{array}{cc} L_{Matting}= & L_{1}\left(\alpha_{i}^{*},\alpha_{i}^{t}\right)+L_{lap}\left(\alpha_{i}^{*},\alpha_{i}^{t}\right)+L_{T}\left(\alpha_{i}^{*},\alpha_{i}^{t}\right)+\\ \; & L_{1}\left(F_{i}^{*},F_{i}^{t}\right)+L_{T}\left(F_{i}^{*},F_{i}^{t}\right), \end{array}$$

where $F_{i}^{*}$ and $F_{i}^{t}$ denote the prediction of the foreground and the ground truth of foreground. Foreground pixels can also be constrained as multi-channel learning to keep the robustness of matting.

### 4.2. Evaluation on HD/SD Datasets

We evaluate the benchmark constructed in RVM [3] on our composite test dataset. More specifically, we select 200 frames as random background images and 100 frames in each video clip and image sample from VM and AIM to build our test dataset.

We compare our method against the state-of-the-art video matting model, RVM; the trimap-based method, FBA; the background-based method, BGM (BGMv2); and the auxiliary-free methods MODNet and PPMatting. Different backbones may interfere with the matting performance, so we conduct the experiments on a variety of backbones, including MobileNetv3, ResNet-50, ResNet-101, and FasterNet. We do not use transformer backbones like MatteFormer [30] in order to save training time and GPU memory.

We evaluate the metric of the matting alpha, $\alpha^{*}$, and the matting foreground, $F^{*}$. For those models that do not predict the $F^{*}$, we use $\alpha^{*}*I$ to matte the foreground from the RGB frame, where *I* denotes the sample frame. The performance of matting $\alpha^{*}$ is evaluated by MAD (mean absolute difference), MSE (mean squared error), Grad (spatial gradient), and Conn (connectivity) for higher level quality comparison, and temporal robustness is evaluated by dtSSD. For the quality of foreground, we only measure pixels where $\alpha^{*}>0$ by MSE and MAD. $F^{*}$ is not measured on VM since the ground-truth foreground frames have obvious noisy edges.

**Figure 4:**
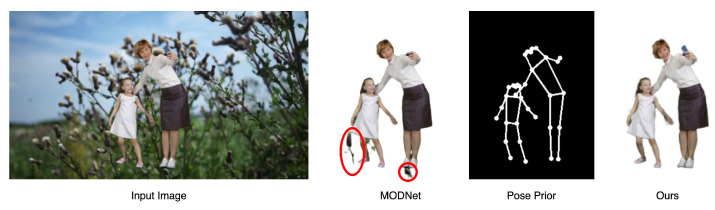
The comparison of matting performance with MODNet and our ControlMatting.

**Figure 5:**
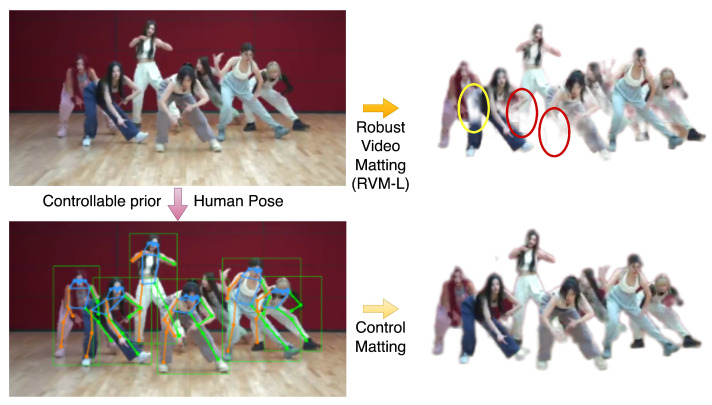
The comparison of the matting performance of RVM-Large and ControlMatting.

**Figure 6:**
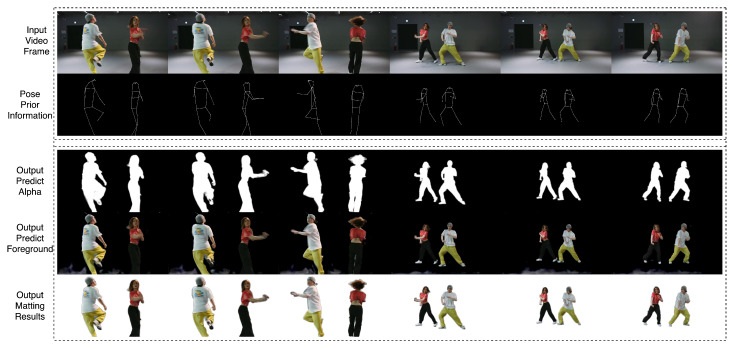
The matting performance of ControlMatting on a two-person dance video.

Table 1 and Table 2 demonstrate the metrics evaluated on the SD (<576 × 480 resolution) test dataset. DeepLabV3 and FBA cannot output edges with sufficient details. BGM and MODNet show bad performance on videos with dynamic backgrounds. RVM with MobileNetv3 and ResNet-50 may output a defect alpha when there are unfocused humans in the background. We show the performance comparison of our model and MODNet in Figure 4. Our model outperforms all of these models on these metrics without a controllable encoder. The controllable encoder mainly improves the matting ability in multi-person scenarios but also optimizes all of the metrics. Table 3 shows the metrics evaluated on the HD (>1920 × 1080 resolution) test dataset. Our detailed refiner decoder is used to obtain better edge detail recovery. Conn is not computed because of the large computation on high-resolution frames, and we reach the SOTA benchmark on all other metrics. In Figure 5, we demonstrate the comparison of our model and RVM-Large in a dynamic ambiguous multi-person frame, which is quite a difficult matting problem, and our model obtains a more successful result. In Figure 6, we show the satisfactory matting performance of ControlMatting with pose prior information for a two-person dance video, in which the frames contain a number of factors that can easily lead to the deterioration of the matting performance, such as dynamic semantic ambiguity discrimination caused by blurring between frames, fine-grained mask extraction from the edge of long hair and clothing, and defect masking caused by hats and shoes with very similar colors to the wall and floor.

### 4.3. Ablation Studies

**Figure 7:**
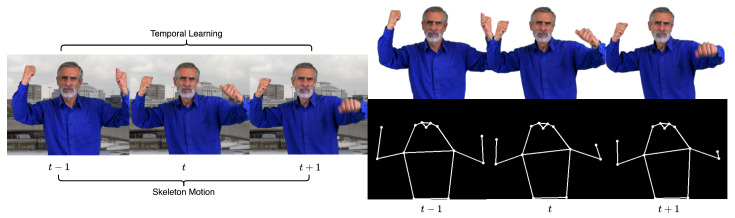
Display the matting performance of consecutive frames using ControlMatting.

Figure 7 displays the matting performance of consecutive frames using our ControlMatting. We compare our baseline and ablation models to confirm the effectiveness of our backbone, controllable encoder, L-ASPP layer, and detailed refiner decoder. Table 4 demonstrates the experimental results of ablation studies and shows three important conclusions. First, our FasterNet* outperforms other backbones applied in past models, and FasterNet obtains awful performance on generative tasks while using its original quadruple downsampling in the first stage. Quadruple downsampling affects the model’s ability to extract edge detail features. The PConv block used in FasterNet* indeed effectively reduces the redundant calculations in channels, and experimental results prove that it is better than the backbone module with the same number of parameters. Second, L-ASPP and the controllable encoder give a significant boost to the metrics on the SD-size test dataset. As a transition layer between the encoder and decoder, the L-ASPP module effectively extracts multi-scale perceptual feature and also plays a unified role in the output features of the basic encoder and conditional encoder. Third, our detailed refiner effectively helps the model handle high-resolution frames. Although only learned in the third stage of training, the auxiliary information provided by the coarse alpha in the front layers of the decoder enables the refiner to quickly and efficiently complete edge details.

The results of ablation experiments confirm the validity of our work, and our final model achieves the best performance on both the SD and HD test datasets. Figure 5 proves that the single-person semantic prior derived from the dataset is not enough to generalize to complex multi-person scenarios, and controlling the auxiliary prior information can overcome this problem. When there are a large number of people in the scene, the performance of RVM will be significantly worse. One possible reason is that the portrait dataset used for training lacks the data of multi-person scenes, resulting in poor results in real-world multi-person videos.

In the ablation experiments, there were no comparative experiments conducted on other existing matting models by inserting the controllable encoder unit or other modules proposed in this paper. Conducting such parallel experiments would require additional parameter settings and adjustments to experimental training strategies, making it difficult to ensure the fairness of comparative experiments. Additionally, most existing matting models have already incorporated other auxiliary modules, such as the detailed semantic learning branch in MODNet. Inserting the model proposed in this paper in parallel may interfere with the feature selection of the decoder and cannot guarantee the stability of the training results. Considering these factors, the ablation experiments in this paper are only conducted on our ControlMatting model. The experimental data from these experiments are sufficient to demonstrate the effectiveness of each module.

**Table 4:** Ablation experiments on the VM dataset.

Backbone	L-ASPP	Detailed Refiner	CE	Size	MAD (1 × 10^3^)	MSE (1 × 10^3^)	Grad	Conn	dtSSD (1 × 10^2^)
MobileNet-v3	-	-	-	512 × 288	6.08	1.47	0.88	0.41	1.36
ResNet-50	-	-	-	512 × 288	5.66	0.92	0.75	0.37	1.32
ResNet-101	-	-	-	512 × 288	5.62	0.91	0.72	0.34	1.29
FasterNet	-	-	-	512 × 288	28.81	17.16	5.35	3.53	2.24
FasterNet*	-	-	-	512 × 288	5.40	0.88	0.71	0.29	1.19
FasterNet*	✓	-	-	512 × 288	5.39	0.88	0.69	0.28	1.17
FasterNet*	✓	-	✓	512 × 288	**5.31**	**0.79**	**0.65**	**0.26**	**1.08**
FasterNet*	✓	-	-	1920 × 1080	5.54	0.91	9.32	-	1.82
FasterNet*	✓	✓	-	1920 × 1080	5.51	0.91	9.25	-	1.68
FasterNet*	✓	✓	✓	1920 × 1080	**5.46**	**0.87**	**9.20**	-	**1.62**

### 4.4. Speed Comparison

The real-time performance of the matting model is also significant for further applications, and Table 5 shows the speed and size comparisons for different models. However, our controllable encoder is an auxiliary subnetwork that occupies independent computing resources to solve the matting problem in multi-person and dynamic scenes, and the size of ControlMatting is larger than RVM and MODNet. Benefiting from the partial convolution operation in PConv, our model saves a lot of channel computing redundancy and can still meet the real-time demand standards required by general applications. Table 5 gives the FPS on the 3840 × 2160 resolution images on an NVIDIA GeForce 3090 with PyTorch1.10 compiling acceleration and JIT optimization. A useful way to speed up the inference is PyNvCodec, which provides full HW acceleration for video processing tasks. We also tested the impact of different deep learning computing frameworks on the resource usage and speed of matting models. Executable file compiled with the C++ version of libtorch can reduce the GPU memory usage of ControlMatting by 40%. At the same time, PyTorch has a phenomenon of first slowing down and then getting fast in the speed measurement experiment, which also limits the further development of model performance.

However, rapid real-time models also exhibit deficiencies when processing real-world videos. Our paper addresses these issues by employing additional controllable encoders and conditional prior information to ensure the model’s matting performance in complex, dynamic videos with multiple individuals. The experimental comparisons with other existing matting models demonstrate improved performance. However, it should be noted that the controllable encoder provides only referential rather than decisive features to the decoder, which ensures the model’s matting performance without using additional conditional prior information, thus achieving faster inference speeds. In practice, when dealing with videos with excessive complexity and multiple occlusions, the model’s performance may still degrade. This phenomenon is attributed to potential errors in the conditional prior information extracted by the controllable encoder, which may misguide the decoder. One solution is to employ larger, more powerful models for extracting conditional prior information, but such approaches are challenging to deploy in real-time scenarios, which remains a primary challenge for future work.

**Table 5:** Comparison of model parameters, speed, GMACs, size, and FPS.

Model	Parameters	Size	GMACs	FPS
DeepLabV3 + FBA	95.68 M	233.3 M	205.77	5.3
BGMv2	5.01 M	19.4 M	8.46	48.5
MODNet	6.49 M	25.0 M	8.80	178.5
RVM	3.75 M	14.5 M	4.57	145.7
RVM-Large	26.89 M	102.9 M	98.68	86.8
Our Model	17.54 M	67.08 M	29.95	93.8
Our Model + CE	32.78 M	125.4 M	52.58	61.5

## 5. Conclusions

In this paper, we propose ControlMatting, a new matting architecture to improve the matting performance on complex multi-person dynamic videos. Compared with the existing matting model based on deep learning, the introduction of controllable prior information effectively solves the problem that it is difficult for a single convolutional network structure to simultaneously learn complex human body semantic prediction and pixel-level edge detail distribution. Matting is an important step in extracting and synthesizing foreground materials in video synthesis tasks, and ControlMatting provides an efficient method for matting multi-person scenes in natural dynamic backgrounds, which can optimize the performance of existing video synthesis tools. Further, we adopt a backbone network with a more significant effect on key feature extraction, a feature aggregation bottleneck layer, and a learnable edge filter module with mask-guided progressive fine-tuning parameters, thus ensuring the temporal robustness of the matting performance and edge integrity. Moreover, these modules can be easily inserted into the existing matting models, and the matting performance in multi-person scenes can be effectively improved without significant changes to the original model, which can greatly facilitate the optimization of video synthesis-related applications. The multi-stage training strategy of jointly iterating the matting dataset and the semantic segmentation dataset also effectively enhances the ability to distinguish fuzzy objects in the background.

Evaluation experiments prove that our method achieves SOTA on mainstream video matting datasets and effectively enhances the semantic discrimination ability for more complex scenes, which has been ignored by existing methods. Although the introduction of conditional priors increases the computational burden, our model can still meet the requirement of real-time operation. In the future, our work will focus on extracting more representative original features in frames and using methods such as hardware acceleration to speed up inference so that the model can be deployed to mobile applications quickly and more conveniently.

## Figures and Tables

**Table 1:** SD-resolution comparison experiments on the VM dataset.

Model	MAD (1 × 10^3^)	MSE (1 × 10^3^)	Grad	Conn	dtSSD (1 × 10^2^)
DeepLabV3	14.47	9.67	8.55	1.69	5.18
FBA	8.36	3.37	2.09	0.75	2.09
BGMv2	25.19	19.63	2.28	3.26	2.74
MODNet	9.41	4.30	1.89	0.81	2.23
VideoMatte	6.06	1.27	1.09	0.42	1.60
RVM	6.08	1.47	0.88	0.41	1.36
RVM-Large	5.66	0.92	0.75	0.37	1.32
Our Model	5.39	0.88	0.69	0.28	1.17
Our Model + CE	**5.31**	**0.79**	**0.65**	**0.26**	**1.08**

**Table 2:** Low-resolution comparison experiments on the AIM dataset.

Model	MAD (1 × 10^3^)	MSE (1 × 10^3^)	Grad	Conn	dtSSD	Fgr (1 × 10^3^)
DeepLabV3	29.64	23.78	20.17	7.71	432	-
FBA	23.45	17.66	9.05	6.05	229	6.32
BGMv2	44.61	39.08	5.54	11.60	269	**3.31**
MODNet	21.66	14.27	5.37	5.23	176	9.51
RVM	14.84	8.93	4.35	3.83	101	5.01
RVM-Large	13.48	4.58	3.95	3.38	98	4.79
Our Model	11.86	4.28	3.73	3.25	94	4.55
Ours + CE	**11.32**	**4.19**	**3.68**	**3.09**	**90**	4.33

**Table 3:** HD-resolution comparison experiments on the VM dataset.

Model	MAD (1 × 10^3^)	MSE (1 × 10^3^)	Grad	dtSSD (1 × 10^2^)
MODNet	11.13	5.54	15.30	3.08
RVM	6.57	1.93	10.55	1.90
RVM-Large	5.81	0.97	9.65	1.78
Our Model	5.51	0.91	9.25	1.68
Ours + CE	**5.46**	**0.87**	**9.20**	**1.62**

## Data Availability

This paper uses open source datasets. For the source, detailed information, and usage methods, please refer to References [56,57,58].
